# Human-like problem-solving abilities in large language models using ChatGPT

**DOI:** 10.3389/frai.2023.1199350

**Published:** 2023-05-24

**Authors:** Graziella Orrù, Andrea Piarulli, Ciro Conversano, Angelo Gemignani

**Affiliations:** Department of Surgical, Medical, Molecular and Critical Area Pathology, University of Pisa, Pisa, Italy

**Keywords:** ChatGPT, machine learning, NLP, problem-solving, AI, Artificial Intelligence

## Abstract

**Backgrounds:**

The field of Artificial Intelligence (AI) has seen a major shift in recent years due to the development of new Machine Learning (ML) models such as Generative Pre-trained Transformer (GPT). GPT has achieved previously unheard-of levels of accuracy in most computerized language processing tasks and their chat-based variations.

**Aim:**

The aim of this study was to investigate the problem-solving abilities of ChatGPT using two sets of verbal insight problems, with a known performance level established by a sample of human participants.

**Materials and methods:**

A total of 30 problems labeled as “*practice problems*” and “*transfer problems*” were administered to ChatGPT. ChatGPT's answers received a score of “0” for each incorrectly answered problem and a score of “1” for each correct response. The highest possible score for both the *practice* and *transfer* problems was 15 out of 15. The solution rate for each problem (based on a sample of 20 subjects) was used to assess and compare the performance of ChatGPT with that of human subjects.

**Results:**

The study highlighted that ChatGPT can be trained in out-of-the-box thinking and demonstrated potential in solving verbal insight problems. The global performance of ChatGPT equalled the most probable outcome for the human sample in both *practice problems* and *transfer problems* as well as upon their combination. Additionally, ChatGPT answer combinations were among the 5% of most probable outcomes for the human sample both when considering *practice problems* and pooled problem sets. These findings demonstrate that ChatGPT performance on both set of problems was in line with the mean rate of success of human subjects, indicating that it performed reasonably well.

**Conclusions:**

The use of transformer architecture and self-attention in ChatGPT may have helped to prioritize inputs while predicting, contributing to its potential in verbal insight problem-solving. ChatGPT has shown potential in solving insight problems, thus highlighting the importance of incorporating AI into psychological research. However, it is acknowledged that there are still open challenges. Indeed, further research is required to fully understand AI's capabilities and limitations in verbal problem-solving.

## 1. Introduction

The use of machine learning (ML) in psychology is becoming increasingly widespread. Large amounts of data generated in psychological studies can be effectively analyzed and interpreted using ML algorithms. Such methods can assist researchers in identifying patterns and relationships in data that are not immediately obvious. ML algorithms can, for example, be used to analyse brain imaging data and identify features associated with various neurological or psychiatric disorders (Orrù et al., 2012, 2021; Ferrucci et al., 2022), or in overlapping futures of chronic conditions (i.e., fibromyalgia: Orrù et al., 2020a) which are particularly frequent (Dell'Osso et al., 2015). ML can be effectively applied in a range of fields such as forensic sciences (malingering: i.e., Sartori et al., 2017; Pace et al., 2019; personality faking-good: i.e., Mazza et al., 2019) and psychological research (Orrù et al., 2020b), amongst others.

New ML models such as large language models (LLM) are purportedly the promoters of a recent “paradigm shift” in artificial intelligence-based (AI-based) language analysis. Indeed, LLMs are sophisticated AI systems that are trained on vast amounts of textual data. These models have the ability to generate human-like language and perform a wide range of language tasks such as translation, question-answering and sentiment analysis, amongst others. These models, like the *Bidirectional Encoder Representations from Transformers* (BERT) and the *Generative Pre-trained Transformer* (GPT) and their chat-based variations (i.e., ChatGPT), have produced previously unheard-of levels of accuracy (Devlin et al., 2018) in most computerized language processing tasks and have recently gained widespread attention. GPT is a language model created by OpenAI. The latest and most advanced language model developed is known as GPT and has been trained on a massive amount of text data from the internet. It can generate human-like text and perform various language tasks such as translation, summarization, question answering and coding. GPT-3 enables a personalized conversation with an AI bot capable of providing detailed responses to questions (prompts) at significant speed. Specifically, GPT-3 is a deep learning autoregressive language model (a simple feed-forward model), that produces human-like text from a set of words given in a specific context. In general terms, the ability of the LLM to mathematically represent words in context is presumably largely responsible for its success.

The Transformer-based model is a specific neural network architecture introduced by Vaswani et al. (2017) in “*Attention is all you need*” (2017) and has become the foundation of many state-of-the-art models in Natural Language Processing (NPL), including GPT-3. The main innovation of transformer architecture is the use of attention mechanisms, which allow the model to selectively focus on different parts of the input during its processing, and thus to understand more effectively the relationships between words and phrases in the input. Specifically, attention allows the association of distant portions of text within a sentence; for example, it enables the understanding that in the sentence “*the boy chasing the horse is fat*,” “*fat*” refers to the boy and not the horse. LLMs are massive neural networks, consisting of billions of parameters, that are trained on vast quantities of text and rely on an attention mechanism. One of the most efficient training systems is the one used in GPT-3, which involves predicting the next word in a sentence; for example, by displaying the sentence “*the dog barks and the cat…*,” it predicts the word “*meows*.”

Fine-tuning is a method used for training a pre-trained transformer-based model on a new dataset, with the aim of adapting it to a specific task. This is achieved by training the model on a smaller dataset that is specific to a certain task, while maintaining the weights from the pre-trained model fixed. This procedure enables the model to employ the knowledge that it has learned from the larger dataset to quickly learn how to perform a new task.

The purpose of the present work was to evaluate the ability of ChatGPT in solving verbal insight problems from two sets of problems, both of which were originally solved by a group of 20 human participants in a study conducted by Ansburg and Dominowski (2000). The aim of this evaluation was twofold: firstly, to determine whether ChatGPT could solve these types of verbal insight problems, which are typically associated with human intelligence and have been previously considered challenging for computers to solve; secondly, to compare ChatGPT's problem-solving abilities to those of humans, as established in the mentioned study.

Overall, the present study sought to assess the potential of ChatGPT as an intelligent tool for problem-solving and to explore the extent to which machine intelligence can match or surpass human intelligence in this domain.

In the sections that follow, the study begins by providing (i) a framework to better understand problem-solving; (ii) a summary of the state-of-the-art classification techniques used in speech contexts and of the transformer architecture, emphasizing the components involved in encoding and decoding; (iii) the major findings are then presented; (vi) finally, the difficulties involved, and potential futures directions are discussed.

### 1.1. Verbal insight problem-solving

To comprehend insight problem-solving, key terms must first be defined. Problem-solving is defined as a set of cognitive processes aimed at transforming a given circumstance into a desirable scenario when there is no clear solution (Mayer and Wittrock, 2006). In other words, it represents the process of finding a solution to a problem or a set of problems that involves the use of different strategies or techniques to overcome obstacles and reach a specific goal, “*when no solution method is obvious to the problem solver*” (Mayer, 1992). There are different approaches to problem-solving, including analytical, creative, and intuitive methods: *analytical methods* involve breaking down the problem into smaller parts and systematically analyzing each part to find a solution (i.e., Polya, 2004); *creative methods* involve generating new and unique ideas to solve the problem; *intuitive methods* involve the use of past experiences and knowledge to inform the problem-solving process. Problem-solving has been thoroughly investigated by cognitive science in general with a number of theories and models being put forward. One of the most influential theories is the “*General Problem Solver*” (GPS) hypothesis of Newell and Simon (1972) which states that problem-solving is a logical and systematic process that adheres to a set of norms and processes. Another important theory is the “*Dual-Process Hypothesis*” (DPT), proposed by Kahneman (2011), which suggests that problem-solving can be achieved through both quick, intuitive processes (System 1) and slow, deliberate processes (System 2). A further problem-solving method is known as “*Insight*” can be defined as the unexpected development of a new concept or a fresh perspective on the issue and it is frequently characterized by an “*Aha!*” moment. Insight problem-solving has been extensively studied by cognitive psychologists and was first described by Wallas (1926) as a four-stage process consisting of preparation, incubation, illumination, and verification.

### 1.2. A brief overview of cutting-edge sequence classification methods in text or speech contests

In end-to-end sequence classification, ML, and specifically deep learning, are becoming increasingly popular. This type of classification requires only a single model to learn all those stages in between initial inputs and the final outputs. There are two basic methods for analyzing sequential data in text or speech; these methods are referred to as Transformers and Recurrent Neural Networks (RNNs).

A transformer-based model, as mentioned above, is a type of neural network architecture introduced by Vaswani et al. (2017). The key innovation of the transformer is the self-attention mechanism, also known as intra-attention, which connects “*different positions in a single sequence to compute a representation of the sequence*” according to the authors. Self-attention allows the model to evaluate the importance of different parts of the input when making a prediction. Reading comprehension, meaningful summarization, and learning task-independent sentence representations have all been effectively implemented with self-attention using transformer architecture, which has been widely employed in a wide range of NPL processing applications (i.e., BERT and GPT-2), serving as the foundation for a number of pioneering models (i.e., Parikh et al., 2016; Petrov et al., 2016). In contrast, RNNs, the predecessors to transformers, process inputs sequentially word after word (i.e., text or speech) and struggle with long-range dependencies (Li et al., 2018). In an RNN, the hidden state of the network at each timestep is a function of the hidden state at the previous timestep and the current input. This means that the prediction at each timestep depends on predictions at all previous timesteps, making it difficult to parallelize computation (Le et al., 2015). RNNs generate a vector from a sequence in order to capture the meaning of an entire sentence, a strategy that performs poorly when dealing with long and complex sentences. In this context, the transformer architecture was specifically designed to overcome this RNN limitation by introducing a self-attention mechanism.

### 1.3. Encoding and decoding components

**Figure 1:**
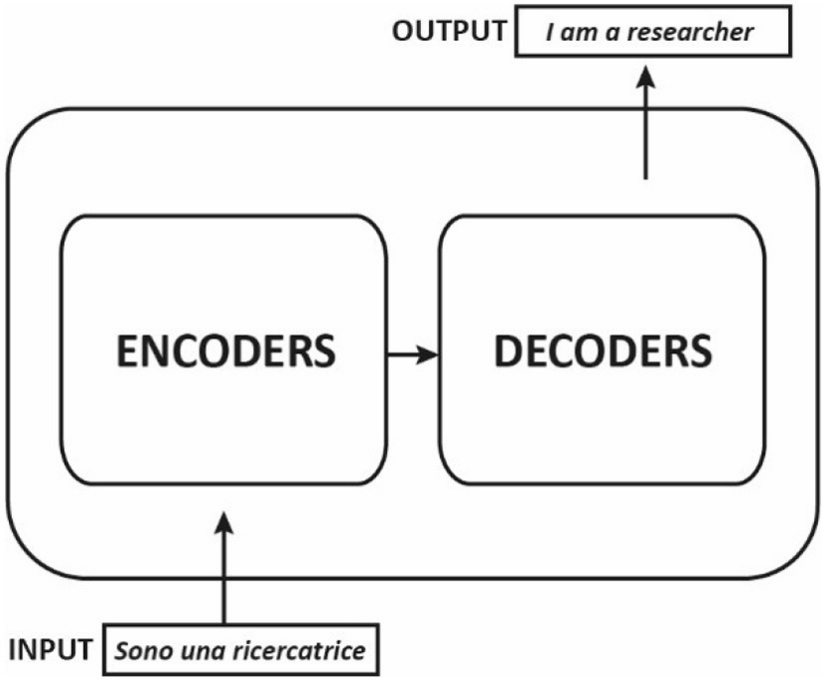
Encoding and decoding components in a machine translation application.

Most competitive neural sequence transduction models, such as those used in NLP tasks, have an encoder-decoder structure. The encoder-decoder structure was first introduced in the paper “*Sequence to Sequence Learning with Neural Networks*” by Sutskever et al. (2014). They suggested employing RNNs for both the encoder and the decoder. This architecture was later improved by the incorporation of the attention mechanism, which allows the decoder to evaluate the importance of different parts of the input when generating the output, as proposed in “*Neural Machine Translation by Jointly Learning to Align and Translate*” by Bahdanau et al. (2014). This structure consists of two main components: an encoder, which processes the input sequence and produces a fixed-length representation, and a decoder, which generates the output sequence based on the fixed-length representation, for example in a machine translation application from Italian to English (Figure 1).

**Figure 2:**
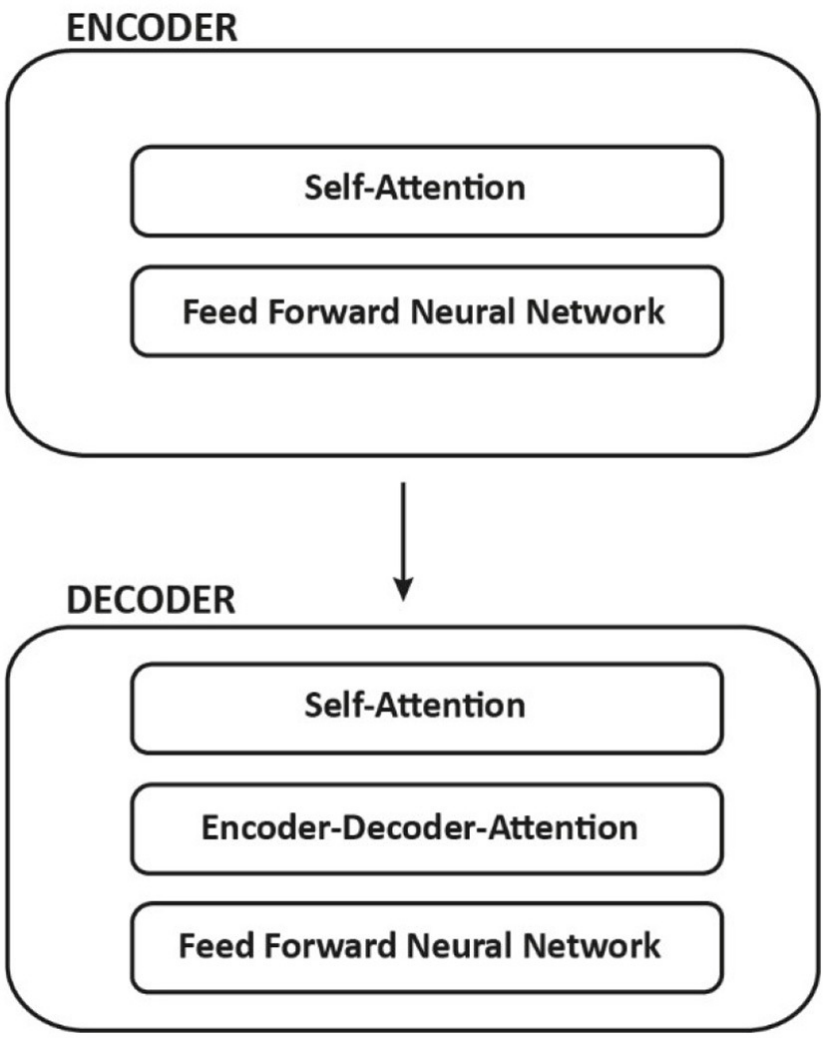
Encoder and decoder structure and sublayers.

Each encoder has the same structure and is made up of two sub-layers: *Self-Attention* and *Feed Forward Network*. Encoder inputs first pass through a self-attention layer, which assists the encoder in looking at other words in the input sentence while encoding a specific word. Self-attention layer outputs are fed into a feed-forward neural network. The same feed-forward network is applied to each position independently. Both layers are also present in the decoder yet between them is an attention layer, the *encoder-decoder attention*, that assists the decoder in focusing on relevant parts of the input sentence such as constituents, dependencies, semantic roles and coreferences, among others (Figure 2).

## 2. Materials and methods

Thirty verbal insight problems were administered to ChatGPT, as listed in the study by Ansburg and Dominowski (2000): the first set of 15 problems was referred to as “*practice problems*,” while the second set of 15 problems was referred to as “*transfer problems*” (see Ansburg and Dominowski, 2000; Appendix A, p. 54–59). The two sets of problems were only used to verify the ability of ChatGPT to solve verbal problem-solving tasks, not to replicate their experimental procedure.

### 2.1. Language-based instructions

Before beginning the administration of verbal insight problems to ChatGPT, a contest was created (known as a prompt in LLM jargon) for ChatGPT with the following short instruction: “*Try to solve this practice problem. The first one is the following*” and the first *practice problem* was provided: “*A farmer in California owns a beautiful pear tree. He supplies the fruit to a nearby grocery store. The store owner has called the farmer to see how much fruit is available for him to purchase. The farmer knows that the main trunk has 24 branches. Each branch has exactly 6 twigs. Since each twig bears one piece of fruit, how many plums will the farmer be able to deliver?*”. In this case ChatGPT failed to solve the problem and the feedback “*The answer provided is not correct*” was provided. It was then fed with “*control instructions*” (CI) (see Ansburg and Dominowski, 2000, Appendix B, p. 59) before solving the first problem again. After the second failure, the ChatGPT was provided with “*strategic instructions*” (SI) (see Ansburg and Dominowski, 2000, Appendix C, p. 60). The CI and SI were listed by Ansburg and Dominowski (2000; p. 59–60). The remaining verbal tasks were then subsequently presented, and relevant feedback was provided to ChatGPT based on the correct/incorrect answer given, such as “*The answer is correct. The correct answer is* + solution” or “*The answer is not correct. The correct answer is* + solution.”

### 2.2. Methods

The ChatGPT (Jan. 9, 2023) was employed. As stated above, a total of 30 problems, labeled “*practice problems*” and “*transfer problems*” (see the study of Ansburg and Dominowski for more information) were administered to ChatGPT. According to the solutions for the *practice problems* and *transfer problems* described by Ansburg and Dominowski (2000), the score “0” was assigned for each problem which ChatGPT answered incorrectly and “1” was attributed for each correct answer. For the two sets of 15 problems, the maximum score attributable was 15. For an exhaustive overview of the administered problems and of human sample and ChatGPT's performance refer to Tables 1, 2. The performance of ChatGPT was compared to that of the human sample by using the solution rate to each problem (Tables 1, 2) wich was derived from Ansburg and Dominowski (2000)'s study based on a sample of 20 subjects. Our intention was to utilize only the stimuli from Ansburg and Dominowski, rather than replicate the study itself.

**Table 1:** Problem-solving tasks: the 15 practice problems and solutions (correct answers), ChatGPT answers, the score attributed (answer accuracy) and solution rate of the human sample.

**Problem-solving tasks**			
**Practice problems**	**Correct answer**	**ChatGPT response**	**Answer accuracy**
(1) A farmer in California owns a beautiful pear tree. He supplies the fruit to a nearby grocery store. The store owner has called the farmer to see how much fruit is available for him to purchase. The farmer knows that the main trunk has 24 branches. Each branch has exactly 6 twigs. Since each twig bears one piece of fruit, how many plums will the farmer be able to deliver?	None, plums do not grow on pear trees	To solve this problem, we need to find the total number of twigs on the pear tree. According to the information given in the problem, the main trunk has 24 branches and each branch has 6 twigs. This means that the total number of twigs is 24 branches ^*^ 6 twigs/branch = ≪ 24^*^6 = 144 ≫ 144 twigs.	0
		Since each twig bears one piece of fruit, the farmer will be able to deliver 144 twigs ^*^ 1 fruit/twig = ≪144^*^1 = 144 ≫ 144 plums. Answer: \boxed{144}.	
**Solution rate: 50%**			
(2) Hardy Pyle was bragging about his church's baseball team. He said, “*Three of our players hit home runs and two of those home runs were hit with the bases loaded. Our guys won 9 to 0 and not a single man crossed home plate*.” How was this possible?	The men were all married, or the players were all female.	*It is possible that Hardy Pyle's church's baseball team won the game 9 to 0 without any men crossing home plate because they scored all of their runs through home runs. If three players hit home runs and two of those home runs were hit with the bases loaded, then the team would have scored at least 5 runs (3 home runs + 2 home runs with the bases loaded = 5 runs). If the team scored a total of 9 runs, then the remaining 4 runs could have been scored through additional home runs or other means such as errors or walks. Therefore, it is possible for a team to win a baseball game 9 to 0 without any men crossing home plate if they score all of their runs through home runs or other means*.	0
**Solution rate: 21.1%**			
(3) In what year did Christmas and New Year's fall in the same year?	They always do.	*It is not possible to determine the year in which Christmas and New Year's fell in the same year based on the information provided in the problem. **Christmas and New Year's always fall in the same year**, as they are consecutive holidays that are celebrated on December 25th and January 1st, respectively. Therefore, every year, Christmas and New Year's fall in the same year*.	1
**Solution rate: 75%**			
(4) How many cubic meters of dirt are in a hole 6 meters long, 2 meters wide and one meter deep?	There is no dirt in a hole.	To find the volume of a rectangular prism (such as a hole), you can use the formula: volume = length ^*^ width ^*^ height. In this case, the hole is 6 meters long, 2 meters wide and one meter deep, so the volume of the hole is 6 meters ^*^ 2 meters ^*^ 1 meter = ≪621 = 12≫12 cubic meters. Therefore, there are 12 cubic meters of dirt in the hole. Answer: \boxed{12}.	0
**Solution rate: 20%**			
(5) There is an ancient invention still used in some parts of the world today that allows people to see through walls. What is it?	Windows	*It is not possible to determine the ancient invention that allows people to see through walls based on the information provided in the problem. There is no such invention that allows people to see through walls. Some forms of technology, such as certain types of radar or thermal imaging cameras, can be used to detect objects or people behind walls or other barriers, but they do not allow people to actually see through the walls. Therefore, it is not possible to accurately answer this problem*.	0
**Solution rate: 65%**			
(6) Captain Frank was out for a walk when it started to rain. He did not have an umbrella and he wasn't wearing a hat. His clothes were soaked yet not a hair on his head got wet. How could this happen?	He was bald	*It is possible that Captain **Frank's hair did not get wet because he was bald**. If a person is bald, they do not have any hair on their head, so it is not possible for their hair to get wet. Therefore, it is possible for Captain Frank's hair to not get wet even though his clothes were soaked in the rain, if he is bald. This is the only explanation that can be inferred from the information provided in the problem*.	1
**Solution rate: 65%**			
(7) Mr. and Mrs. Plum have six daughters and each daughter has one brother. How many people are in the Plum family?	There are nine people in the Plum family.	*The Plum family consists of Mr. Plum, Mrs. Plum, and their six daughters. Each daughter has one brother, so there are an additional 6 brothers in the family. In total, there are 6 daughters + 6 brothers + Mr. Plum + Mrs. Plum = ≪6 + 6 + 1 + 1 = 14≫14 people in the Plum family. Answer: \boxed{14}*.	0
**Solution rate: 19%**			
(8) If you drop a rock, would it fall more rapidly through water at 40 degrees Fahrenheit or 20 degrees Fahrenheit? Explain your answer.	40 degrees because at 20 water is frozen	***A rock would fall more rapidly through water at 40 degrees Fahrenheit than at 20 degrees Fahrenheit**. The density of a substance is a measure of the mass of the substance per unit volume. The density of water decreases as the temperature increases, which means that warm water is less dense than cold water. This means that a rock will fall more slowly through warm water than through cold water because the warm water is less dense and offers more resistance to the movement of the rock. **At 40 degrees Fahrenheit, the water is warmer and less dense than at 20 degrees Fahrenheit, so the rock will fall more rapidly through the water at 20 degrees Fahrenheit than at 40 degrees Fahrenheit**. This is because the denser, colder water at 20 degrees Fahrenheit will offer more resistance to the movement of the rock, causing it to fall more slowly through the water*.	1
**Solution rate: 55%**			
(9) Shadow opened the door to Dr. Apple's office and surveyed the scene. Dr. Apple's head lay on his desk in a pool of blood. On the floor to his right lay a gun. There were powder burns on his right temple indicating that he was shot at close range. On his desk was a suicide note, and in his right hand the pen that had written it. Shadow noted that death had occurred in the past hour. All of a sudden Dr. Apple's wife burst into the office and screamed “*My husband's been shot!*” She ran toward the body and saw the note and cried, “*Why would he want to kill himself?*” Shadow replied “*This was no suicide, it is a clear case of murder*.” How does Shadow know?	The gun would be the last thing in his right hand not the pen if it were suicide.	*Shadow knows that the death of Dr. Apple was a case of murder because there are powder burns on his right temple, indicating that he was shot at close range. A suicide victim typically shoots themselves at close range, so the presence of powder burns suggests that the shooter was in close proximity to the victim when the shot was fired. In a suicide, the victim is the one who fires the shot, so there would not be any powder burns on their temple. Therefore, the presence of powder burns on Dr. Apple's temple suggests that someone else was present when the shot was fired and that Dr. Apple was murdered*.	0
**Solution rate: 65%**			
(10) A man who lived in a small town in the United States married 20 different women of the same town. All are still living and he never divorced any of them. In this town, polygamy is unlawful; yet, he has broken no law. Can you explain this?	The man is a minister or justice of the peace.	*It is not possible to explain how the man in the small town in the United States has married 20 different women without breaking the law on polygamy, as polygamy is the practice of having more than one spouse at the same time, and it is unlawful in the United States. It is not legal for a person to be married to multiple spouses at the same time in the United States. Therefore, it is not possible for the man in the small town to have married 20 different women without breaking the law. The information provided in the problem is contradictory and cannot be reconciled*.	0
**Solution rate: 10%**			
(11) Can a man marry his widow's sister? Explain your answer.	No, the man would be dead.	***It is not possible for a man to marry his widow's sister, as the man would have to be deceased in order for his spouse to be a widow**. A widow is a woman whose spouse has died, and a person cannot marry their own spouse if they are deceased. Therefore, it is not possible for a man to marry his own widow's sister*.	1
**Solution rate: 55.6%**			
(12) Sid Shady was working for a large construction company that was very concerned about employee theft. Someone tipped the company security that Shady was the man to watch. Each night, he passed through security with a wheelbarrow full of scrap lumber, discarded electrical wires and chunks of concrete. The security guards checked the contents daily, but could find nothing of value. What was Shady stealing?	He was stealing wheelbarrows.	*It is not possible to determine what Sid Shady was stealing based on the information provided in the problem. The security guards checked the contents of Shady's wheelbarrow each night and found nothing of value, so it is possible that Shady was not stealing anything at all. The company may have been mistaken in its suspicion of Shady or the person who tipped off the company may have provided incorrect or misleading information. Without additional information, it is not possible to accurately answer this problem*.	0
**Solution rate: 57.9%**			
(13) At 7 a.m., a train moving 90 kph leaves Montreal heading for Toronto. At 8 a.m., a train running 110 kph leaves Toronto heading for Montreal. Which train will be closer to Montreal when they meet?	When they meet, the trains will be at the same spot.	***When the two trains meet, they will be the same distance from Montreal**. This is because the trains are traveling in opposite directions and will cover equal distances from their respective starting points. At 7 a.m., the train leaving Montreal is 90 kph and at 8 a.m., the train leaving Toronto is 110 kph. If the trains are traveling in opposite directions, **they will cover equal distances from their respective starting points, so when they meet, they will be the same distance from Montreal***.	1
**Solution rate: 28.6%**			
(14) A woman said to her husband: “*This morning, one of my earrings fell into my coffee. Even though my cup was full, the earring did not get wet.”* How could this be true?	It fell into coffee grounds.	***Yes, it is possible that the woman's earring fell into the coffee grounds rather than the coffee itself, which would explain why the earring did not get wet even though the cup was full**. When coffee is brewed, the water passes through the coffee grounds and the liquid coffee is produced. **The coffee grounds remain in the filter or coffee pot and do not get wet**. If the earring fell into the coffee grounds rather than the liquid coffee, it would not get wet. This is a possible explanation for the woman's statement that her earring fell into her coffee but did not get wet*.	1
**Solution rate: 50%**			
(15) Barney Dribble is carrying a pillow case full of feathers. Hardy Pyle is carrying three pillow cases the same size as Barney's, yet Hardy's load is lighter. How can this be?	Hardy's pillow cases are empty	*Yes, **if Hardy's pillow cases are empty, they would be lighter than Barney's pillow case filled with feathers**, which would explain how Hardy's load is lighter even though he is carrying three pillow cases the same size as Barney's. I'm glad that I could help clarify this for you. If you have any other questions, please don't hesitate to ask*.	1
**Solution rate: 60%**			
		Total score	7/15

**Table 2:** Problem-solving tasks: the 15 transfer problems and solutions (correct answers), ChatGPT answers, the score attributed (answer accuracy) and solution rate of the human sample.

**Problem-solving tasks**			
**Transfer problems**	**Correct answer**	**GPT's response**	**Answer accuracy**
(1) Professor Bumble was driving along in his old car when suddenly it shifted gears by itself. He paid no attention and kept on driving. Why wasn't he concerned?	He was driving an automatic	*The **Professor Bumble was not concerned when the car shifted gears by itself because the car was most likely an Automatic gear shifting car**. In an automatic car, gears are changed automatically by the car itself without the need for manual shifting from the driver. This means that it's normal for the car to shift gears by itself and the driver doesn't need to take any action, Professor Bumble would expect this behavior from the car. Therefore, in this case, he didn't pay attention and kept on driving because he was not concerned that anything was wrong*.	1
**Solution rate: 40%**			
(2) Which would be worth more, a pound of $10 pure gold coins or half a pound of $20 pure gold coins; or would they be worth the same amount? Explain your answer.	A pound of gold is always worth more than half a pound of gold.	*Both a pound of $10 pure gold coins and half a pound of $20 pure gold coins would be worth the same amount. Even though the $20 pure gold coins are worth twice as much as the $10 pure gold coins, half a pound of $20 pure gold coins would have half the weight as a pound of $10 pure gold coins. Since the value of the gold coins is based on their weight and gold's value is per ounce, the weight of the coins will determine the value of it. And since the weight of both options is the same, the value would be the same as well. Therefore, the answer is that both options would be worth the same amount, regardless of the face value of the coins*.	0
**Solution rate: 45%**			
(3) Dee Septor, the famous magician, claimed to be able to throw a ping-pong ball so that it would go a short distance, come to a dead stop, and then reverse itself. He also added that he would not bounce the ball against any object or tie anything to it. How could he perform this feat?	He could throw it straight up	*Dee Septor, the magician, could perform this feat by using a hidden magnet. He could throw a ping-pong ball that has a small magnet embedded inside it, and as it comes to a stop, the magnet would be attracted to a hidden magnet that he has positioned in the opposite direction. This would cause the ball to reverse its direction without bouncing against any object or having anything tied to it*. *Another way, he could use air pressure to change the direction of the ball, by blowing air to the ball as it comes to a stop, it will change its direction. This is a common trick used by magicians to make it look like the ball is changing direction on its own*. *Both of these explanations involve the use of hidden and unseen elements to create the illusion of the ball reversing itself, and are not actually using the ball's properties to reverse it, they are just hidden tricks to make the audience think that the ball is reversing its direction*.	0
**Solution rate: 36.4%**			
(4) A prisoner was attempting to escape from a tower. He found in his cell a rope that was half long enough to permit him to reach the ground safely. He divided the rope in half, tied the two parts together, and escaped. How could he have done this?	He unraveled the rope and tied the two pieces together	*The prisoner could have done this by tying the two halves of the rope together, forming a knot and making it whole again. By doing this he could have doubled the strength of the rope, and this would have made it long enough to safely reach the ground*. *Another way, he could have used the two halves of the rope as two separate ropes to make a ladder by tying one end of each rope on a different level of the tower, and then climb down each step of the ladder. This way he can combine the two halves to reach the ground*. *In this problem, the prisoner used the limited resources he had at his disposal, his intelligence and creativity to overcome the obstacle and find a solution to escape*.	0
**Solution rate: 40%**			
(5) Uriah Fuller, the famous super-psychic, can tell you the score of any baseball game before it starts. What is his secret?	The score is always zero to zero before a game starts	*As a language model AI, I do not have the ability to access current or past information about any baseball games, because my training data goes until 2021 and my browsing ability is disabled*. *But to answer your question, Uriah Fuller the super-psychic would have some kind of ability to predict the future, such as precognition, clairvoyance or divination. He could use this ability to know the outcome of a baseball game before it starts*. *It is important to note that while some people may claim to have psychic abilities, there is no scientific evidence to support the existence of these abilities, and Uriah Fuller's “super-psychic” abilities are also fictional and not real*.	0
**Solution rate: 30%**			
(6) Two mothers and two daughters were fishing. They managed to catch one big fish, one small fish and one fat fish. Since only three fish were caught, how is it possible that they each took home a fish?	One of the mothers was also a daughter	*This question is a classic riddle, the solution to this problem is not a straightforward one. **The key to the answer is to realize that the mothers are the two daughters' mother, and the daughters are the two mothers' daughters**. **So, they are a family of three: a grandmother, a mother, and a daughter**. With this information, it becomes clear that each of them is able to take a fish home, because they are three different people, not four*. *The riddle is describing a scenario that is not intended to be taken literally, instead, it is meant to present a problem in a way that requires a different way of thinking, to find the solution*.	1
**Solution rate: 30%**			
(7) Calendars made in England do not show Lincoln's Birthday, of course. Do these calendars show the fourth of July? Explain your answer.	Yes, July always has a fourth day	*The answer is no, calendars made in England do not show the fourth of July. The reason is that the fourth of July is Independence Day in the United States, and it is not a national holiday in England. Lincoln's Birthday is also not a national holiday in England, as it is in the United States. This means that neither Lincoln's Birthday nor the 4th of July are typically included on calendars made in England. As such, both holidays are specific to the United States and not recognized in England*.	0
**Solution rate: 42.1%**			
(8) A dealer in antique coins got an offer to buy a beautiful bronze coin. The coin had an emperor's head on one side and the date 544 BC stamped on the other. The dealer examined the coin and realized that it was a fake. How did he know the coin was phony?	Christ was had not been born, so a coin from that time would not be marked BC	*The dealer knew the coin was a fake because the date 544 BC was stamped on the other side. **This is not possible because the concept of “BC” (Before Christ) was not created until several centuries later, and the concept of using “BC” as a notation to indicate a date before the birth of Jesus Christ was not widely used until the Middle Ages**. Additionally, the concept of a calendar based on the birth of Jesus was not established until several centuries after the death of Jesus. This means that the concept of “BC” would not have existed at the time period represented by the date 544 BC. Therefore, the dealer realized that the coin was a fake because the date stamped on it was not accurate for the time period it purported to be from*.	1
**Solution rate: 35%**			
(9) There is a town in Northern Ontario where 5% of all the people living in the town have unlisted phone numbers. If you selected 100 names at random from the town's phone directory, on average, how many of these people selected would have unlisted phone numbers?	Zero, they would not be listed in the directory	*If 5% of all the people in the town have unlisted phone numbers, that means for every 100 people, 5 of them will have unlisted phone numbers. So, if you select 100 names at random from the phone directory, on average, 5 of those people selected will have unlisted phone numbers*.	0
**Solution rate: 30%**			
(10) It is estimated that the earth weighs 6 sextillion tons. How much more would the earth weigh if one sextillion tons of concrete and stone were used to build a wall?	It would weigh the same since all materials are taken from the original weight of the earth	*A sextillion is a very large number, it is equivalent to 1 followed by 21 zeroes (10^∧^21). Since the wall is made of concrete and stone, the weight of the wall will be added to the earth's weight. If one sextillion tons of concrete and stone were used to build the wall, the earth would weigh 7 sextillion tons, which is one sextillion tons more than its current weight*.	0
**Solution rate: 31.8%**			
(11) A young boy turned off the light in his bedroom and managed to get into bed before the room was dark. If the bed is ten feet from the light switch and he used no wires, strings or other contraptions to turn off the light, how did he do it?	It was still daylight	*The answer is that the light was turned off remotely. The boy is able to turn the light off before the room is dark because he is using a remote control or some other form of wireless technology to turn off the light instead of physically moving to the switch and turning it off*.	0
**Solution rate: 35%**			
(12) While on safari in the wild jungles of Africa, Professor Quantum woke one morning and felt something in the pocket of his shorts. It had a head and tail, but no legs. When Quantum got up he could feel it move inside his pocket. Quantum, however, showed little concern and went about his morning rituals. Why such a casual attitude toward the thing in his pocket?	It was a coin	*Answer: The **object in question was a coin***.	1
**Solution rate: 36.8%**			
(13) Professor Bumble, who is getting on in years, is growing absent minded. On the way to a lecture 1 day, he went through a red light and turned down a one-way street in the wrong direction. A policeman observed the entire scene but did nothing about it. How could Professor Bumble get away with such behavior? Montreal when they meet?	He was on foot	*Answer: **He was walking** not driving*	1
**Solution rate: 30%**			
(14) A giant inverted steel pyramid is perfectly balanced on its point. Any movement of the pyramid will cause it to topple over. Underneath the pyramid is a $100 bill. How would you remove the bill without disturbing the pyramid?	Burn or tear the dollar bill	*You could use a long, thin tool, such as a chopstick or a pencil, to slide the bill out from underneath the pyramid without physically touching the pyramid itself. This way, the pyramid remains balanced and undisturbed*.	0
**Solution rate: 40%**			
(15) Hardy Pyle was washing windows on a high-rise office building when he slipped and fell off a sixty-foot ladder onto the concrete sidewalk below. Incredibly, he did not injure himself in any way. How was this possible?	He was on the bottom rung of the ladder	*The ladder was leaning against a wall on the first floor, Hardy was not at a high altitude when he fell*.	0
**Solution rate: 31.8%**			
		Total score	5/15

Putative between-set differences were estimated both for ChatGPT and for the human sample (*n* = 20). In the former case significance was assessed using Fischer Exact Test, while in the latter by performing a Wilcoxon rank sum test on the solution rates. Unless otherwise stated, descriptive statistics are presented as median and interquartile ranges (25 percentile-75 percentile).

#### 2.2.1. Probability distributions of the possible outcomes for the human samples on each set of problems

For both problem sets, the probability distribution related to each possible total score (i.e., number of correct answers) on the human sample (see Supplementary material 1) was estimated. Note that the most likely outcome is the score showing the higher occurrence probability.

The *k*^*th*^ score probability was obtained as follows:

- The set of all possible answer combinations was first identified, producing the desired score. As an example, let us consider a score of three (three correct answers out of fifteen): such a score can be obtained by various combinations of answers, their number equalling the binomial coefficient $\left(\frac{15}{3}\right)$. Given a set of *n* items (problems/answers, *n* = 15), for each *k*^*th*^ score (*k*^*th*^ ranging from 0 to 15), all answer combinations composed by *k* correct answers were identified (the total number of combinations $C_{k^{th}}\left(n,k\right)$ is equal to: $\frac{n!}{k!\left(n-k\right)!}$).
- As a second step, the probability associated with the *k*_*i*_ combination was computed, ($k_{i}\in k^{th}$ as: $p_{k_{i}}=\prod_{j\in k_{i}}p_{j}^{*}\prod_{j\in \left(n-k_{i}\right)}\left(1-p_{j}\;\right)$
- Finally, the total probability of obtaining a total score of *k*^*th*^ was obtained as the sum of the related probabilities over the entire set of combinations ($C_{k^{th}}$): $p_{k^{th}}=\underset{k_{i}\in \;C_{k^{th}}}{\sum }p_{k_{i}}$.

For each set of problems, the most likely outcome in the human sample was compared (i.e., the total score showing the highest occurrence probability) to the total score obtained by ChatGPT. It should be noted that this test accounts for the similarities between humans and ChatGPT global performance on a set of problems *irrespective* of paired differences/similarities related to the performance on the single problems within the set.

#### 2.2.2. Problem-wise associations between human population solution rates and ChatGPT performance

Among possible answer combinations leading to a total score equal to that obtained by ChatGPT, those corresponding to ChatGPT answer combinations were identified and subsequently compared to the occurrence probability of all other answer patterns leading to the same total score. This procedure was applied both problems.

#### 2.2.3. Probability distributions of the possible outcomes and associations between human population solution rates and ChatGPT performance on the pooled dataset

The same analyses were then conducted by pooling together the two problem sets. This choice was motivated by the utility of having a general view of ChatGPT performance as compared to that of the human population, independently of the problem type and the related solution strategy. The appropriateness of the pooling in based on the fact that the number of problems is balanced across the two sets (15 problems each).

## 3. Results

The performance of ChatGPT on practice and transfer problems are shown in Tables 1, 2, respectively. Each table displays the problems assigned to ChatGPT as well as the proper solutions (correct answers), ChatGPT answers, the score obtained (answer accuracy) and the solution rate of the human sample.

The performance of ChatGPT was subsequently compared with those of a sample of individuals (*n* = 20) in the two sets of problems.

ChatGPT answered correctly to seven problems out of fifteen in the case of the *practice problems*, and to five out of fifteen in the case of *transfer problems*: the ChatGPT performance between-set difference was not significant (Fischer Exact Test, *p* < 0.72, see Supplementary material 2). Human sample solution rates were respectively 0.55 (0.23–0.64) on the *Practice* set, and 0.35 (0.30–0.40) on the *transfer* set. While the solution rate was higher for the former set as compared to the latter, the between-set difference was not significant (Wilcoxon rank sum test, z = 1.29, *p* < 0.20, see Supplementary material 3).

**Figure 3:**
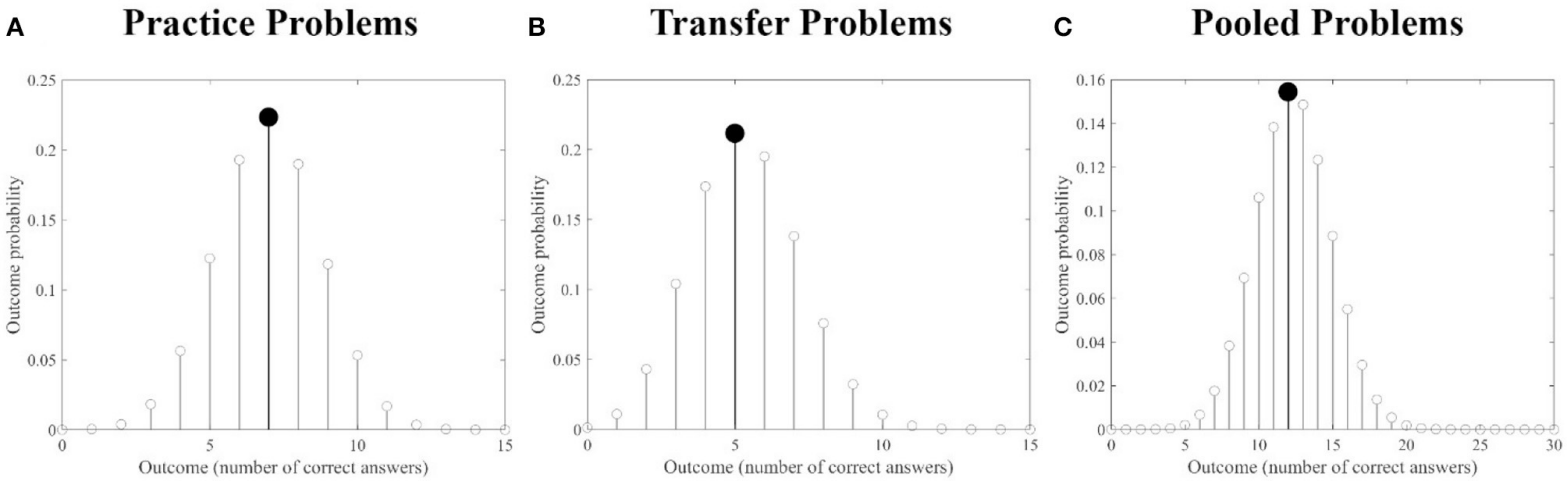
Human sample outcome probabilities: outcome probabilities for each possible total score (i.e., number of correct answers, range 0–15) are presented for the *Practice* set **(A)**, the *Transfer* set **(B)** and the pooled set [**(C)**, *Practice* + *Transfer* Problems]. In each plot, the outcome with the highest probability is highlighted in black. Notably, the total score with the highest probability is equal to the performance of ChatGPT for both for each set of problems and for the pooled set.

Notably, for both sets of problems, ChatGPT performance (i.e., the number of correct answers) was equal to the total score of the human sample showing the highest occurrence probability, as clearly apparen from Figures 3A, B.

**Figure 4:**
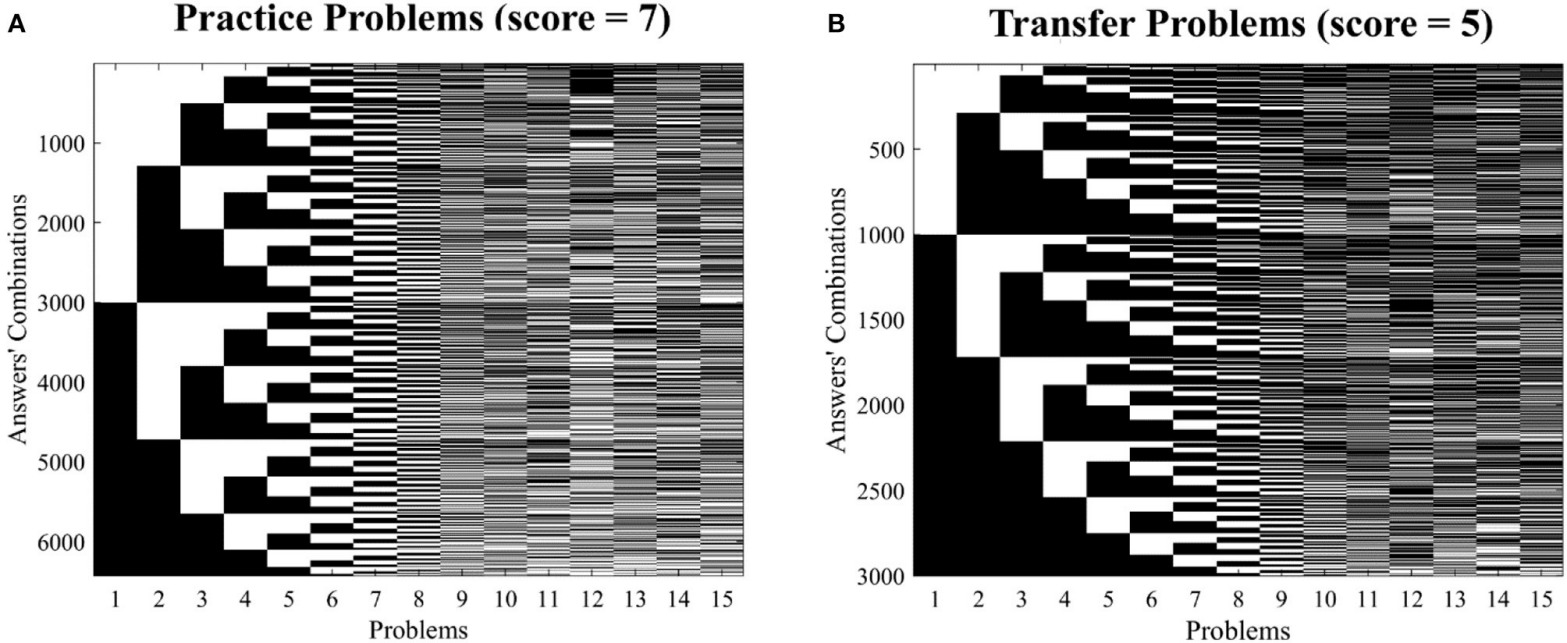
Answer patterns equalling the ChatGPT total score: for each set of problems, all possible answer combinations leading to a score equal to that obtained by ChatGPT are presented [Practice set, **(A)** and Transfer set, **(B)**]. In each matrix, rows correspond to all possible combinations and columns to the answers. Each matrix element identifies a possible answer within a combination (black = correct, white = wrong). The matrix related to the pooled set is not presented as the number of possible combinations exceeded 86,000,000 and as such the image would have been unintelligible.

For each problem set, all answer combinations leading to the total ChatGPT score was identified (see Figures 4A, B) and their occurrence probability on the human sample estimated (see Figures 3A, B). For each set, the ensemble of combinations included that of ChatGPT:

First set of problems (*practice problems*): [0, 0, 1, 0, 0, 1, 0, 1, 0, 0, 1, 0, 1, 1, 1].

Second set of problems (*transfer problems*): [1, 0, 0, 0, 0, 1, 0, 1, 0, 0, 0, 1, 1, 0, 0].

**Figure 5:**
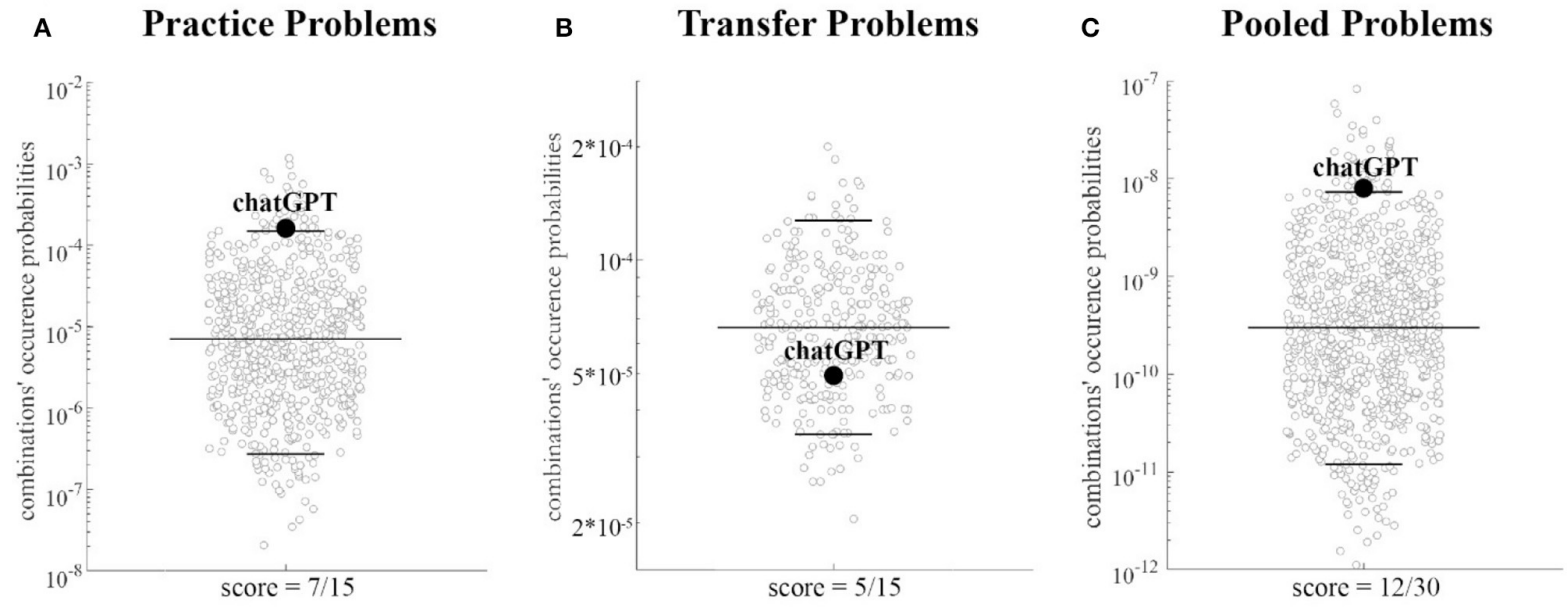
Distributions of answer combination probabilities equalling ChatGPT score: for each set of problems the distribution of probabilities associated with all possible answer combinations leading to a total score equal to that obtained by ChatGPT is presented using a scatterplot. The 5th, 50^th^, and 95th percentiles of the distribution are highlighted by black horizontal lines, whereas the probability associated with answer combinations equalling that of ChatGPT is identified by a black dot. Note that for ease of visualization, in each plot a down sampled number of combinations and probabilities are presented using a logarithmic scale (y-axis). **(A–C)** refer, respectively to *Practice Problems, Transfer Problems*, and Pooled Problems sets.

When considering the *Practice Problems*, the ChatGPT combination occurrence probability (*p* ≅ 1.52e-04), was observed as being above the threshold identifying the 5% percentile of those combinations showing the highest occurrence probability in the human sample (*p* ≅ 1.50e-04, see Figure 5A, and Supplementary material), thus indicating an association between the human sample problem by problem performance and that of ChatGPT. However, the ChatGPT combination occurrence probability on the *Transfer* set (*p* ≅ 5.01e-05) neared the 27th percentile of the occurrence probability distribution, thus resulting in a high unlikely solution for the human population (i.e., no association between the problems correctly solved by ChatGPT and those with a higher solution rate for the human sample, see Figure 5B).

### 3.1. Pooled problems set

As a further step the two sets of problems were pooled together. This choice was supported by the following three main points:

1. The number of problems was balanced across the two sets (15 problems each).
2. ChatGPT performance on the Practice set was not significantly different from that obtained on the Transfer set (Fischer Exact Test, *p* < 0.72, see Supplementary material 1).
3. The median solution rate of the human population on the Practice set was not significantly different from that obtained on the transfer set (Wilcoxon rank sum test, z = 1.53, *p* < 0.13, Supplementary material 2).

When considering the pooled datasets, ChatGPT performance (i.e., number of correct answers = 12), was again equal to the total score of the human population showing the highest occurrence probability (Figure 3A). The ChatGPT combination (*p* ≅7.61e-09) was above the threshold identifying the 5% percentile of those patterns showing the highest occurrence probability in the human sample (*p* ≅ 7.35e-09, see Figure 5C), thus indicating an association between the human sample problem by problem performance and that of ChatGPT, including when considering the entire dataset.

## 4. Discussion and conclusions

In the current study, ChatGPT was provided with two sets of verbal insight problems, namely one set of *practice* and one of *transfer* problems (each set consisting of 15 problems, for a total of 30 problems). The score was assigned based on the accuracy of the answers provided by ChatGPT. The study's findings revealed that the global performance of ChatGPT was equal, as apparent from Figure 3, to the one showing the highest occurrence probability in the human sample: this finding is consistent for the *practice, transfer* and pooled problems sets. These results indicate that ChatGPT performance on both tasks (and on the pooled tasks), were completely in line with those of the average human subject, indicating that it performed similarly to humans. Moreover, both when considering the *practice* and the pooled problem sets, the ChatGPT answers' combination occurrence probability was above the threshold identifying the 5% percentile of those combinations (producing the same total score), showing the highest occurrence probability in the human sample. This was not the case for the *transfer problems* set.

In general terms, LLMs are unquestionably highly competent at making connections and the research presented demonstrates how such connections may be used to complete tasks which were once thought to be impossible. Indeed, LLMs, such as ChatGPT-3, are neural networks that have undergone training to forecast the most probable verbal output (i.e., word/sentence) based on a certain order of words. They make predictions by identifying the word with the strongest association (or probability). It is worth noting that associationism, first introduced as one of the earliest theories in psychology (James, 1890, 2007) waned in popularity when the rise of other theoretical frameworks such as behaviorism and cognitive psychology demonstrated that associationism could not fully explained the intricacy of human language production.

An issue that has sparked widespread debate in the cognitive science community is whether LLMs truly have problem-solving abilities, or such abilities are the result of a deep understanding of the problem. In this context, our findings suggest the possibility that LLMs could employ associationism, thus drastically reducing the number of tasks that complex associational models are unable to perform. Some LLMs, like ChatGPT-3, are able to complete tasks in line with the average human ability and certainly are advanced associators. From this perspective, this calls into question theories which may have led cognitive psychology to rule out associationism., of note this concept was also highlighted by a pre-print paper by Loconte et al. (2023).

It is therefore evident that the number of tasks that an associator is unable to solve is gradually reducing and future research will have to identify limits that cannot be pushed any further as LLMs become ever more competent.

While this study sheds light on the “behavior” of ChatGPT when dealing with verbal problems, it presents some limitations: (i) the size of the sample representative of humans (*n* = 20) to which ChatGPT was compared, was relatively small, and as such, additional testing is required to validate the results herein presented; (ii) the study examined the performance of ChatGPT using only a single version of the model. From this perspective, it would be beneficial to replicate the current study with more recent and/or advanced versions of the model in order to verify whether there has been any improvement; (iii) finally, the study only examined ChatGPT performance on verbal insight problems; it would be of utter interest to investigate how the model performs on other types of problems or tasks.

In conclusion, while this study provides some evidence that ChatGPT performance on verbal insight problems, is similar to those of an average human subject, it is important to recognize its limitations and to continue exploring both the potential and limitations of the model in future studies. Additional research may be carried out in order to expand on the methods and findings presented in this study, allowing for a more comprehensive understanding of the capabilities and limitations of ChatGPT and other LLMs.

## Data availability statement

The data analyzed in this study is subject to the following licenses/restrictions: The dataset used and analyzed during the current study is available from the corresponding author upon reasonable request. Requests to access these datasets should be directed to graziella.orru@unipi.it.

## Author contributions

GO: conceived the experiment, designed the experimental task, and drafted the manuscript. GO and AP: contributed to data acquisition, data analysis, and writing the final version of the manuscript. All authors: data interpretation. All authors revised the manuscript critically and gave final approval of the version to be published.
