# Supplementary material for: Human-like problem-solving abilities in large language models using ChatGPT
Source: Front Artif Intell. 2023 May 24;6:1199350. doi: 10.3389/frai.2023.1199350 (PMC10244637; doi:10.3389/frai.2023.1199350)
Supplement: Supplementary file 1 [file Data_Sheet_1.docx]

Supplementary Material

Human-like problem-solving abilities in Large Language Models using ChatGPT

**Orrù Graziella, Andrea Piarulli, Ciro Conversano, Angelo Gemignani**

*** Correspondence:** Corresponding Author: [graziella.orru@unipd.it](mailto:graziella.orru@unipd.it)

# Occurrence Probability of each possible outcome (total score) on a probems’ set.

When considering the human sample, for each set and problem, only the solution rate averaged over twenty individuals was given (i.e., the solution of each single individual was not available). However, based on the average solution rates, one can determine the probability of occurrence of each possible outcome: for a set of 15 problems the possible outcome ranges from 0 to 15 correct solutions.

For each outcome *k* (number of correct solutions), the occurrence probability can be estimated as the sum of the probabilities related to each combination of k correct answers. As a working example let us consider the probability of obtaining 7 correct answers in the human sample, the same score obtained by ChatGPT (see Table A).

**Table A. Practice Problems**: the human solutions rates, the series of answers given by ChatGPT and another exemplary series of answers leading to the same score *k* = 7 are reported (0 = wrong, 1 = correct). SR stand for Solution Rate.

|  | ***Practice Problems* (*k* = 7)** | | | | | | | | | | | | | | |  |
| --- | --- | --- | --- | --- | --- | --- | --- | --- | --- | --- | --- | --- | --- | --- | --- | --- |
|  | **1** | **2** | **3** | **4** | **5** | **6** | **7** | **8** | **9** | **10** | **11** | **12** | **13** | **14** | **15** | |
| **Human Sample (SR)** | 0.50 | 0.21 | 0.75 | 0.20 | 0.65 | 0.65 | 0.19 | 0.55 | 0.65 | 0.10 | 0.56 | 0.58 | 0.29 | 0.50 | 0.60 | |
| **chatGPT** | 0 | 0 | 1 | 0 | 0 | 1 | 0 | 1 | 0 | 0 | 1 | 0 | 1 | 1 | 1 | |
| **exemplary**  **pattern** | 0 | 1 | 0 | 1 | 1 | 0 | 1 | 0 | 0 | 1 | 1 | 0 | 1 | 0 | 0 | |

The probability of obtaining a total score of 7 correct answer following chatGPT pattern can be estimated as:

$$p_{chatGPT = (1-p\left( 1 \right))(1-p\left( 2 \right))\boldsymbol{p(3)}(1-p\left( 4 \right))(1-p\left( 5 \right))\boldsymbol{p}\left( \boldsymbol{6} \right)\left( 1-p\left( 7 \right) \right)\boldsymbol{p(8)}(1-p\left( 9 \right))\left( 1-p\left( 10 \right) \right)\boldsymbol{p}\left( \boldsymbol{11} \right)\left( 1-p\left( 12 \right) \right)\boldsymbol{p}\left( \boldsymbol{13} \right)\boldsymbol{p}\left( \boldsymbol{14} \right)\boldsymbol{p(15)}}$$

The probability of obtaining a total score of 7 correct answer following the exemplary series of answers from Table A is instead:

$$p_{other = \left( 1-p\left( 1 \right) \right)\boldsymbol{p}\left( \boldsymbol{2} \right)\left( 1-p\left( 3 \right) \right)\boldsymbol{p}\left( \boldsymbol{4} \right)\boldsymbol{p}\left( \boldsymbol{5} \right)\left( 1-p\left( 6 \right) \right)\boldsymbol{p(7)}(1-p\left( 8 \right))(1-p\left( 9 \right))\boldsymbol{p(10)p}\left( \boldsymbol{11} \right)\left( 1-p\left( 12 \right) \right)\boldsymbol{p}\left( \boldsymbol{13} \right)(1-p\left( 14 \right))(1-p\left( 15 \right))}$$

# ChatGPT performance: between-set comparison

ChatGPT performance on the Practice Problems set was compared to the one obtained on the Transfer Problems set, using Fischer Exact Text.

**Table B. ChatGPT contingency table:** for each set of problems (15 problems *per* set), the number of correct and wrong answers are reported.

|  | **Correct Answers** | **Wrong Answers** |
| --- | --- | --- |
| **Practice Problems** | 7 | 8 |
| **Transfer Problems** | 5 | 10 |

ChatGPT performance on the two sets did not significanlty differ one from another (p < 0.72, Odds-Ratio = 1.75).

# Human Sample solution rates: between set comparison

# Putative solution rates differences between Practice and Transfer problems sets were assessed for the human sample (n = 20) by means of Wilcoxon rank sum test.


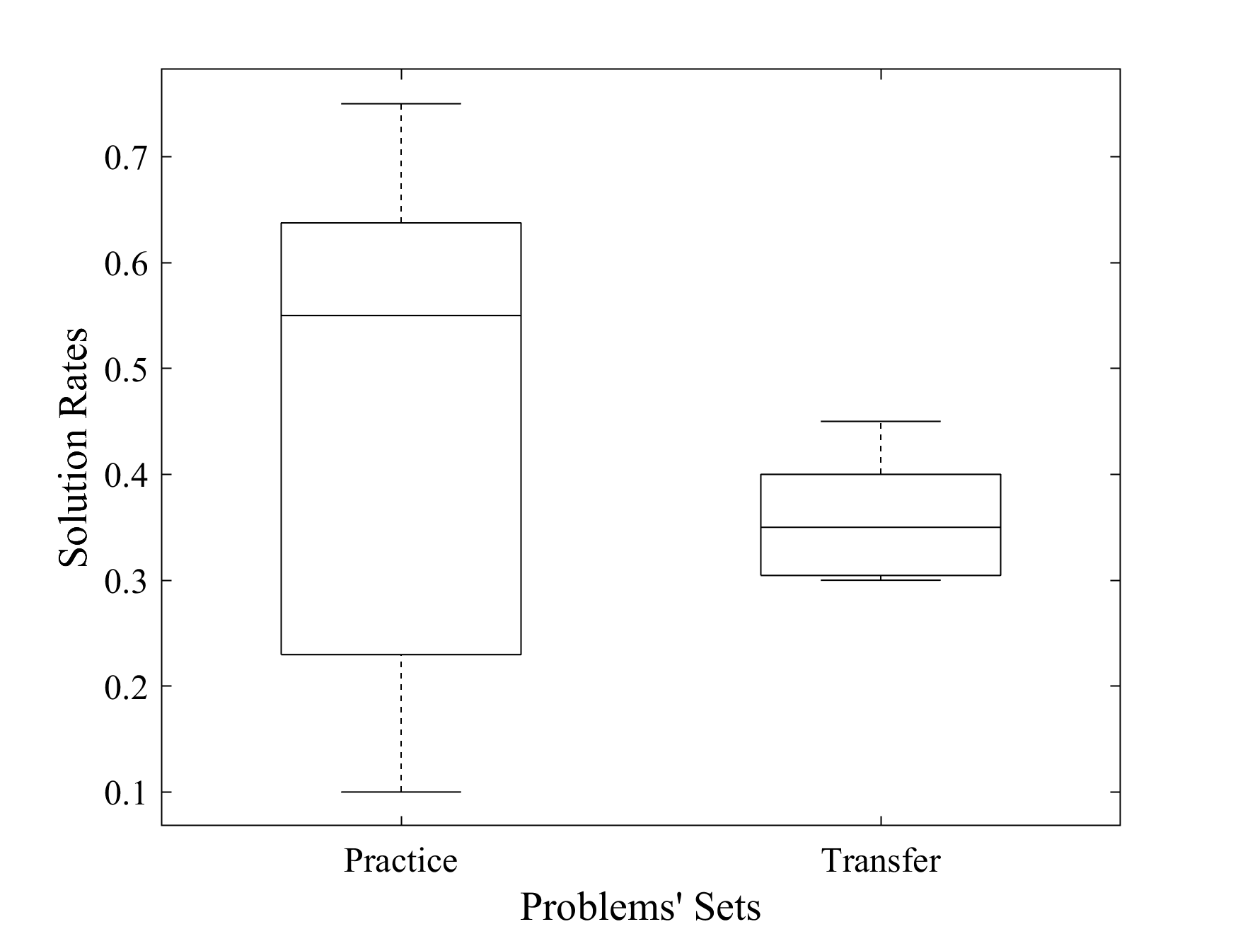


**Figure A. Solution Rates Boxplots:** For each set of problems the boxplot representing the solution rate descriptive statistics is presented.

No significant between-set difference was found between Practice and Transfer solution rates (p < 0.13, z = 1.53), even if the median solution rate of the former set was higher than that of the latter (0.55 versus 0.35).
